# Fast and systematic genome-wide discovery of conserved regulatory elements using a non-alignment based approach

**DOI:** 10.1186/gb-2005-6-2-r18

**Published:** 2005-01-26

**Authors:** Olivier Elemento, Saeed Tavazoie

**Affiliations:** 1 Lewis-Sigler Institute for Integrative Genomics, Princeton University, Princeton, NJ 08544, USA

## Abstract

We describe a powerful new approach for discovering globally conserved regulatory elements between two genomes. The method is fast, simple and comprehensive, without requiring alignments. Its application to pairs of yeasts, worms, flies and mammals yields a large number of known and novel putative regulatory elements. Many of these are validated by independent biological observations, have spatial and/or orientation biases, are co-conserved with other elements and show surprising conservation across large phylogenetic distances.

## Abstract 2

The authors describe a powerful approach for discovering globally conserved regulatory elements between two genomes that does not require alignments. Its application to pairs of yeasts, worm, flies and mammals, yields a large number of known and novel putative regulatory elements, many of which show surprising conservation across large phylogenetic distances.

## Background

One of the major challenges facing biology is to reconstruct the entire network of protein-DNA interactions within living cells. A large fraction of protein-DNA interactions corresponds to transcriptional regulators binding DNA in the neighborhood of protein-coding and RNA genes. By interacting with RNA polymerase or recruiting chromatin-modifying machinery, transcriptional regulators increase or decrease the transcription rate of these genes. Transcriptional regulators bind specific DNA sequences upstream, within or downstream of the genes they regulate, and a large number of experimental and computational studies are aimed at locating these sites and understanding their functions (for example [1,2]). The increasing availability of whole-genome sequences provides unprecedented opportunities for identifying binding sites and studying their evolution. The strong conservation of functional elements (binding sites, protein-coding genes, noncoding RNAs, and so on) across even distantly related species should make it possible to predict these functional elements and prioritize them for experimental validation. The few large-scale comparative genomics approaches for finding transcriptional regulatory elements have so far relied mostly on detecting locally conserved motifs within global alignments of orthologous upstream sequences [3,4]. Although very powerful and straightforward, these approaches cannot be used when upstream regions are very divergent or have undergone genomic rearrangements. For example, aligning the mouse and puffer fish orthologous upstream regions would be very difficult, because of the great reduction that the puffer fish intergenic regions have undergone [5]. Also, global alignments cannot be used when the positions of regulatory elements within functionally conserved promoter regions have been scrambled, for example through genomic rearrangements. Also, global alignment-based approaches often generate an overwhelming number of predictions because of the basal conservation between the genomes under study. To reduce the number of predictions, multiple global alignments of upstream sequences from several related species have been used, yielding many new candidate binding sites [3,4]. However, multiple (more than two) closely related genome sequences are not always available; moreover, by focusing only on regulatory elements that are conserved between several genomes, these approaches might miss elements that are conserved in more local areas of the phylogenetic tree.

Here we describe a simple and efficient comparative approach for finding short noncoding DNA sequences that are globally conserved between two genomes, independently of their specific location within their respective promoter regions. Our method, which we call FastCompare, is based on a principle that we have termed 'network-level conservation' [6], according to which the wiring of transcriptional regulatory networks should be largely conserved between two closely related genomes.

Our previous attempts at using network-level conservation relied on Gibbs sampling to find candidate regulatory elements [7]. However, Gibbs sampling and related algorithms are not fully appropriate in this context, because of the low density of actual binding sites in pairs of orthologous upstream regions. Moreover, these algorithms are non-deterministic, relatively slow, and rely on sequence sampling, which makes them likely to miss many regulatory elements. While our previous approach was successful at predicting a large fraction of functional regulatory elements in the relatively small yeast genome, analyzing larger and more complex metazoan genomes requires faster and more exhaustive algorithms. Here, we use a faster, simpler and more comprehensive approach for detecting conserved and probably functional regulatory elements using the network-level conservation principle. FastCompare allows comprehensive exploration of the conserved - but not aligned - motifs between two genomes, while retaining a linear time complexity. We apply our approach to a large number of species, including yeasts, worms, flies and mammals, and describe some of the most conserved known and unknown regulatory elements within these genomes. We also show how this approach may help reconstruct part of the transcriptional network and reveal some of its associated constraints. Finally, we show that a large number of predicted motifs are conserved within and across different phylogenetic groups.

## Results

In the following sections, pairs of closely related species are termed phylogenetic groups. We applied FastCompare to the four following phylogenetic groups: yeasts (*Saccharomyces cerevisiae *and *S. bayanus*), worms (*Caenorhabditis elegans *and *C. briggsae*), flies (*Drosophila melanogaster *and *D. pseudoobscura*) and mammals (*Homo sapiens *and *Mus musculus*). For each phylogenetic group, we describe some of the most interesting, known and novel, predicted regulatory elements. For each of these regulatory elements, we perform independent validation using gene expression data, chromatin immunoprecipitation (IP) data, known motifs and data from several biological databases (Gene Ontology (GO)/MIPS, TRANSFAC), and show that the most globally conserved predicted regulatory elements are strongly supported by these independent sources.

### Yeasts

**Figure 1:**
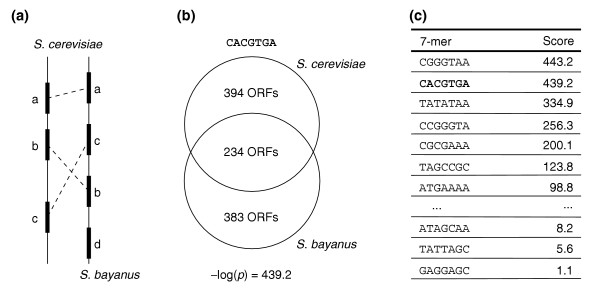
Overview of the FastCompare approach. **(a) **Determination of orthologous pairs of ORFs, and extraction of the associated upstream regions (data not shown). **(b) **For each *k*-mer (here CACGTGA), determination of the sets of ORFs that contain it in their upstream regions, in each species separately. The conservation score (hypergeometric *p*-values to assess the overlap between both sets) is then calculated. **(c) **Ranking of all *k*-mers on the basis of their conservation scores.

The average nucleotide identity between *S. cerevisiae *and *S. bayanus *upstream regions is approximately 62% [4] (similar to the identity between human and mouse upstream regions) and divergence times are estimated between 5 and 20 million years [4]. The number of ortholog pairs between *S. cerevisiae *and *S. bayanus *is 4,358 (see Materials and methods). We chose to analyze 1 kb-long upstream regions, because most of the known transcription factor binding sites in *S. cerevisiae *are located within this range [8]. Using FastCompare, we calculated a conservation score for all possible 7-, 8- and 9-mers on the corresponding 8.6 megabase-pairs (Mbp) of sequences and sorted each list separately according to conservation score (see Figure 1; the raw sorted lists are available on our website [9]). On a typical desktop PC, this analysis took approximately 5 minutes (for example, the entire set (8,170) of 7-mers was processed in 35 seconds).

#### Distribution of conservation scores

**Figure 2:**
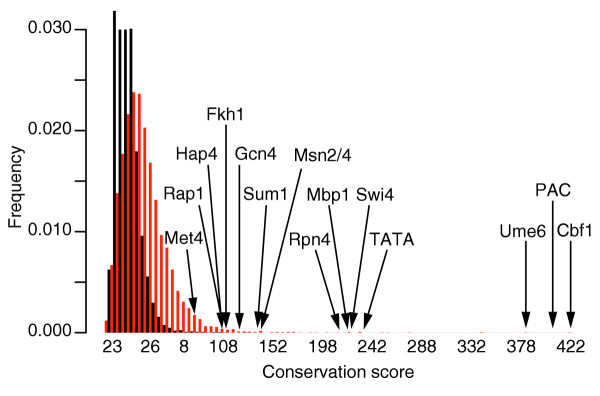
Distributions of conservation scores for actual (red) and randomized (black) data obtained when applying FastCompare to *S. cerevisiae *and *S. bayanus*. Both distributions were constructed using bin sizes of 5. The top portion of the figure is not shown for the purpose of presentation. The distributions show that high conservation scores are unlikely to be obtained from randomized data. Also, a large number of 7-mers on the tail of the distribution correspond to experimentally verified transcription-factor-binding sites in yeast.

As described in Materials and methods, conservation scores are calculated for all *k*-mers (with fixed *k*), and are relative measures of network-level conservation for these *k*-mers (the higher the conservation score, the more conserved the corresponding *k*-mer). We first describe the distribution of conservation scores for all 7-mers. As shown in Figure 2, the distribution of conservation scores has a very long tail and many 7-mers on the tail correspond to well known regulatory elements in *S. cerevisiae *(see below for a detailed description of these sites). To verify that such high conservation scores could not be obtained by chance, we generated randomized sequences as described in Materials and methods and re-ran FastCompare on these sequences. The corresponding distribution of conservation scores is shown on Figure 2 and clearly shows that the high conservation scores corresponding to known regulatory elements are extremely unlikely to arise by chance.

#### Validation using independent biological data

**Figure 3:**
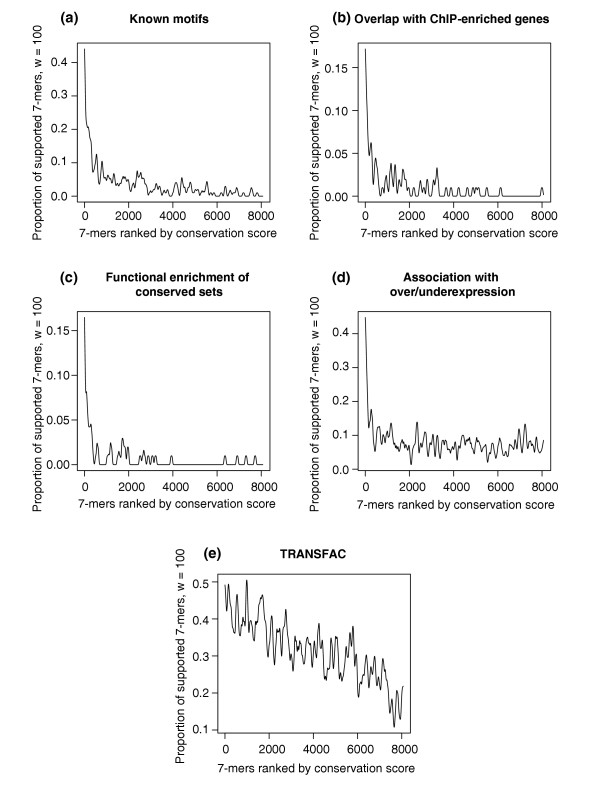
Proportions of 7-mers supported by different types of independent biological data (**(a) **known motifs, **(b) **chromatin-IP, **(c) **functional enrichment, **(d) **under/overexpression, **(e) **TRANSFAC; windows of size 100 were used to construct the figures, see Materials and methods) as a function of the conservation score rank, obtained when applying FastCompare to *S. cerevisiae *and *S. bayanus*. (a-e) strongly indicate that the frequency of support increases with conservation score as calculated by FastCompare.

We used various independent sources of biological data to demonstrate that *k*-mers with the highest conservation scores are likely to be functional. For a given *k*-mer, we define the 'conserved set' as the set of ORFs corresponding to the overlap between the two sets of orthologous ORFs containing at least one exact match to the *k*-mer in their upstream regions (see Materials and methods). We found that conserved sets defined for the highest-scoring 7-mers are significantly enriched with genes whose upstream regions contain occurrences of known motifs in yeast (Figure 3a), significantly enriched with genes whose upstream regions were shown to be bound by known transcription factors *in vivo *(Figure 3b), and significantly enriched in at least one MIPS functional category (Figure 3c). We also show that the number of 7-mers found upstream of over- or underexpressed genes in at least one microarray condition increases with the conservation score (Figure 3d) and that the number of 7-mers matching at least one TRANSFAC consensus also increases with the conservation score (Figure 3e). Altogether, these data provide strong and independent evidence that our method identifies functional yeast regulatory elements by giving them a high conservation score.

Closer examination of Figure 3a-d shows that the 400 highest-scoring 7-mers are most strongly supported by independent data. Therefore we retain them for further analysis and, when possible, replace them by 8-mers and 9-mers with higher conservation scores and also add the high-scoring 8-mers and 9-mers without high-scoring substrings, as described in Materials and methods. This processing yields 398 *k*-mers (*k *= 7, 8 and 9).

Then, for each of these 398 *k*-mers, we determine the optimal window within the initial 1 kb which maximizes the conservation score (see Materials and methods); we then re-evaluate the functionality of each of the 398 *k*-mers with the independent biological information described above, using the new conserved sets. The full information for the 398 *k*-mers is available at [9].

#### Known regulatory elements

Using known transcription factor binding site motifs, genome-wide *in vivo *binding data, functional annotation and literature searches, we found at least 27 different known transcription factor binding sites among the 398 highest scoring *k*-mers. These regulatory elements, along with their support from independent biological data, are shown in Table 1. Some of the best-known binding sites are represented several times within the 398 top scoring *k*-mers, in the form of slightly distinct or overlapping sequences (see [9]). Note also that we use very stringent criteria for identifying known binding sites among our predictions. When we matched our predictions to the known motifs published in [4] (regular expressions), we predicted 42 out of 53 known motifs (Kellis *et al*. [4] predict exactly the same number of motifs, and essentially the same motifs, but using multiple alignments of four yeast genomes).

Among the 27 different known regulatory elements returned by FastCompare, several (Swi4, Mbp1, Sum1/Ndt80, Fkh1/2) are involved in regulating the yeast cell cycle. The other known sites are also involved in fundamental biological processes in yeast: amino-acid metabolism (Cbf1, Gcn4), meiosis (Ume6), rRNA transcription (PAC and RRPE), proteolytic degradation (Rpn4), stress response (Msn2/Msn4) and general activation/repression (Rap1, Reb1). As described in Materials and methods, our approach also handles gapped motifs. Thus, the binding sites for Abf1, a chromatin reorganizing transcription factor (CGTNNNNNNTGA), and Mcm1, a factor involved in cell-cycle regulation and pheromone response (CCCNNNNNGGA), were also identified as very high-scoring patterns and strongly supported by independent information (known motifs and chromatin immunoprecipitation).

When we used the same independent biological data to evaluate the 400 highest-scoring 7-mers obtained on randomized data, we found only three known binding sites (RRPE, FKH1 and BAS1).

Several known binding sites are not found among the 398 top-scoring *k*-mers, perhaps because their transcriptional network has undergone extensive rewiring since the speciation of the two yeasts, or because the corresponding transcription factors regulate few genes. In some cases, the presence of several known sites (clearly identified in terms of independent data) among the full set of 7-mers argues in favor of the rewiring hypothesis. For example, the binding site for the Rcs1 transcription factor, TGCACCC, only appears at the 1,883rd position within the list of ranked 7-mers. Despite its lack of conservation, this site is strongly backed by independent biological information: it is identified as a known motif, it is found in 33 microarray conditions, and its conserved set is significantly enriched in genes annotated with homeostasis of metal ions (*p *< 10^-5^), which is the known function for Rcs1 [10]. Similarly, the known binding sites for the Ace2/Swi5 and Hsf1 transcription factors were clearly identified (in terms of independent data) within the complete list of 7-mers, but not among the 398 highest scoring *k*-mers.

#### Positional constraints

**Figure 4:**
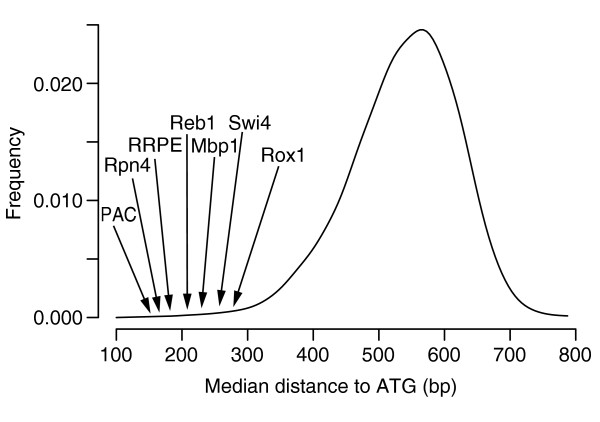
Distribution of median distances to ATG of all 7-mers, obtained when applying FastCompare to *S. cerevisiae *and *S. bayanus*. For each 7-mer, a median distance to ATG was calculated using the positions of matches upstream of *S. cerevisiae *genes within the conserved set for this 7-mer. The 8,170 median distances were then binned into 20-bp bins, and the resulting histogram was smoothed using a normal kernel. The median distances for several known binding sites in *S. cerevisiae *are also indicated (see Table 1).

It is now known that functional regulatory elements can be positionally constrained, relative to other regulatory elements or to the start of transcription [7,11,12]. To assess whether some of the predicted regulatory elements are positionally constrained in yeast, we calculated the median distance to ATG for the conserved sets of each of the 398 *k*-mers and independently built the distribution of median distances to ATG for all 7-mers as described in Materials and methods (the distribution is shown in Figure 4) and found *d*_0.025 _= 350 and *d*_0.975 _= 680. In other words, a median distance to ATG of less than 350 or higher than 680 should each arise by chance with only a 2.5% probability. Among the 398 most conserved *k*-mers, more than a fifth (86) have their median distance below 350 (*p *< 10^-52^), while only seven have a median distance greater than 680. A closer examination reveals that a few known sites are particularly constrained. For example, the binding sites for Reb1, PAC, TATA, Swi4, Rpn4, RRPE and Mbp1 are found to be situated relatively close to the start of translation, with a median distance to ATG between 150 and 300 bp. Some of these constraints were also found to be good predictors of gene expression in a recent study [11] (for RPN4, PAC and RRPE, for example). In contrast, binding sites for Met4, Ume6, Hap4, Rap1, Ino4 and Ste12 are found to be situated at a greater median distance, between 400 and 500 bp from ATG.

#### Novel predicted regulatory elements

We found many novel motifs among our highest-scoring predictions. For example, we found two strongly conserved motifs, AGGGTAA (rank 17) and TGTAAATA (rank 31), which are situated relatively close to ATG (with a median distance to ATG of 349 and 378.5 bp, respectively) and more often in upstream regions than in coding regions (with ratios of 1.95 and 1.83, respectively). Interestingly, TGTAAATA also has a statistically significant 5' to 3' orientation bias (binomial *p*-value < 10^-7^). However, neither of the two putative sites is supported by independent biological data. Additional expression data may help define their biological role. Other sites, such as CAGCCGC or GCGCCGC are found upstream of over- or underexpressed genes in many microarray conditions (15 and 6, respectively). While these two sites are similar to the canonical Ume6-binding site, the latter was not found in any microarray conditions (as none of the microarray experiments we used is related to meiosis, the biological process which Ume6 is known to be involved in), suggesting that the two sites are bound by other factors.

#### Comparing closer and more distant yeast species

We repeated the same analysis on distinct pairs of yeast species other than *S. cerevisiae*/*S. bayanus*. We first compared *S. cerevisiae *and *S. paradoxus *(a much closer relative of *S. cerevisiae*) and found 15 of the 27 known motifs we obtained when comparing *S. cerevisiae *and *S. bayanus *(results are available at [9]). We also compared *S. cerevisiae *with *S. castellii*, which is a more distant relative within the *Saccharomyces *phylogenetic group. *S. castelli *is interesting in that its upstream regions cannot be globally aligned with those of *S. cerevisiae*, because of extensive sequence divergence [3]. We also found 15 of the 27 known motifs found in the *S. cerevisiae*/*S. bayanus *comparison (results at [9]), although they were different from the *S. cerevisiae/S. paradoxus *conserved motifs. Interesting similarities and differences in conservation were revealed when comparing the known motifs discovered in each comparison. For example, the PAC, RRPE and Mbp1 motifs were found within the highest-scoring *k*-mers in all three comparisons, hinting at the conserved role of the corresponding proteins. However, the Reb1-binding site, which was found to be highly conserved between *S. cerevisiae *and *S. bayanus *(rank 1), is much less conserved between *S. cerevisiae *and *S. castelli *(rank 230). This argues for extensive rewiring in the Reb1 transcriptional network in the lineage that led to *S. castelli*.

#### Motif interactions

To discover interactions between regulatory elements, we searched for co-conservation of pairs of high-scoring predicted regulatory elements, as described in Materials and methods. Not surprisingly, the most conserved interaction is between RRPE (AAAAATTTT) and PAC (CTCATCGC), with a median distance *D *= 22 bp [11,13]. We also find that the Cbf1-binding site (CACGTGA) is strongly co-conserved with the Met4-binding site (CTGTGGC), and that these two sites are separated by a short distance (*D *= 44.5) in *S. cerevisiae*. Indeed, it has been shown that the binding of Cbf1 in the vicinity of a very similar sequence (AAACTGTG) enhances the DNA-binding affinity of a Met4-Met28-Met31 complex for this sequence [14], and that the median distance between the above Cbf1 and Met4 sites is small [15].

Many of the predicted interactions have not yet been experimentally studied. For example, we found that the highest scoring Reb1 motif (CGGGTAA) is significantly co-conserved with both the highest scoring RRPE motif (AAAAATTTT) and the highest scoring PAC motif (CTCATCGC), with a short median distance between the two sites in both cases (*D *= 38 and *D *= 63.5, respectively). The Reb1/RRPE interaction was also discovered independently as a good predictor of expression [11]. We also found that Reb1 interacts with the Cbf1 motif (CACGTGA), also at a short median distance (*D *= 30). An interesting interaction between RRPE and an unknown motif, TGAAGAA, displays a conserved set strongly enriched in translation (p < 10^-11^), while RRPE alone is more strongly enriched in rRNA transcription (p < 10^-14^). The full sorted list of interactions is available at [9].

### Worms

In contrast to yeast, relatively little is known about *cis*-regulatory sequences in *C. elegans*. There is a dramatically greater complexity of transcriptional regulation in multicellular organisms. Indeed, transcription factors in multicellular organisms regulate cohorts of genes in different tissues and at different times during development [16]. *C. elegans *promoter regions often contain many domains of activation/repression and, as a result, are much larger than those in yeast.

We applied FastCompare to the genomes of *C. elegans *and *C. briggsae*, two worms that diverged about 50-120 million years ago [17]. The number of orthologous open reading frames (ORFs) between these two species is 13,046 and here we have only considered 2,000 bp upstream regions. It takes approximately 11 minutes for FastCompare to process the corresponding 50 Mbp of sequences and calculate a conservation score for all 7-, 8- and 9-mers on a typical desktop PC.

#### Validations

**Figure 5:**
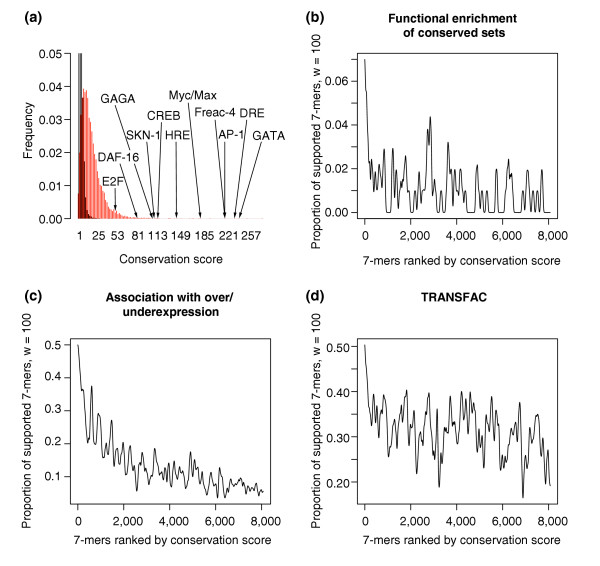
Validation of the conservation scores obtained when applying FastCompare to *C. elegans *and *C. briggsae*. **(a) **Distributions of conservation scores for actual (red) and randomized (black) data, showing that high conservation scores are unlikely to be obtained by chance. Conservation scores for some known regulatory elements are also indicated. Both distributions were constructed using bin sizes of 5, and the top portion of the figure is not shown for the purpose of presentation. **(b-d) **Proportion of 7-mers supported by different types of independent biological data (using windows of size 100, see Materials and methods) as a function of the conservation score rank, obtained when applying FastCompare to *C. elegans *and *C. briggsae*. (b-d) indicate that the frequency of support increases with conservation score as calculated by FastCompare.

The distribution of conservation scores for all 7-mers shows that high conservation scores are unlikely to be obtained by chance (Figure 5a). As shown in Figure 5a, many known regulatory elements fall on the tail of the distribution. We then used functional categories, over- or underexpression, and TRANSFAC motifs to assess the ability of FastCompare to predict functional regulatory elements. Figure 5b-d shows that support for the highest-scoring *k*-mers by functional enrichment, expression and TRANSFAC strongly increases with conservation score. We have only retained the 400 highest-scoring 7-mers, which are particularly well supported by independent biological information as shown in Figure 5b,c. Starting from these 400 highest-scoring 7-mers, we obtain 437 *k*-mers (*k *= 7, 8 or 9) using the procedure described in Materials and methods.

#### Known regulatory elements

As shown in Table 2, at least 15 distinct known binding sites in *C. elegans *and other metazoan organisms were identified among the 437 predicted regulatory elements.

One of the most conserved is TGATAAG, the binding site for the GATA factors, a family of regulators controlling intestinal development (see [18] for review). Another motif returned by FastCompare, GTGTTTGC, corresponds to the binding site for the forkhead-related activator-4 (Freac-4) [19]. Note that this motif is also compatible with the PHA-4-binding site (published consensus: T[AG]TT[GT][AG][CT] [20]), present in the upstream regions of pharyngeal genes [20] (PHA-4 is also a member of the forkhead family of transcription factors). FastCompare also returned TGTCATCA, the known binding site for the SKN-1 transcription factor (published consensus [AT][AT]T[AG]TCAT). In *C. elegans*, SKN-1 is known to initiate mesendodermal development by inducing expression of the GATA factors MED-1 and MED-2 (required for mesendodermal differentiation in the EMS lineage) [21].

The GAGA-factor binding site (AGAGAGA) was also found as a highly conserved pattern. GAGA repeats in upstream regions have been shown to be functional in *C. elegans *in at least two separate studies [22,23]. At least one GAGA-binding protein has been identified in *D. melanogaster*, and is assumed to create nucleosome-free regions of DNA, thus allowing additional transcription factors to bind those regions [24]. However, the ortholog of this protein has not yet been identified in *C. elegans *[24].

We also found CAGCTGG, a site known to be bound by the myogenic basic helix-loop-helix (bHLH) family of transcription factors (in worms, flies and mammals) and AP-4 transcription factors (in mammals) [25,26] (published consensus CAGCTG [27-29]). The homolog of human AP-4 was found to be ubiquitously expressed in *D. melanogaster *and a *C. elegans *homolog has also been identified [25]. FastCompare returned GTAAACA, the known binding site for the DAF-16 transcription factor (published consensus GTAAACA [30,31]). DAF-16, a FOXO-family transcription factor, was shown to influence the rate of aging of *C. elegans *in response to insulin/insulin-like growth factor-1 signaling [31,32].

Searching for gapped motifs found few strongly conserved sites. However, when searching for 8-mers with a 5-bp gap, we found that TGGCNNNNNGCCA, the known binding site for nuclear factor I (NFI) [33], had a score comparable to those of the highest-scoring *k*-mers.

Several of the *C. elegans *sites returned by FastCompare and shown in Table 2 are known to be functional transcription factor binding sites in other species. For example, TGACTCAT, identical to the AP-1-binding site [34], is known to be bound in yeast (by Gcn4), *Drosophila *[35], mouse and human (see [36] for a review).

FastCompare also returns the CACGTGG motif, which is the binding site for the Myc/Max complex, a family of bHLH transcription factors [37]. Among the top-scoring motifs in Table 2, we also find AAGGTCA, the hormone response element (HRE), bound by several transcription factors in human, mouse, fruit fly and silkworm (published consensus [CT]CAAGG[CT]C[AG] [38,39]); TGACGTC, the cAMP response element (published consensus TGACGTCA [40]); CCCGCCC, the binding site for the mammalian Sp1 transcription factor (known consensus CCCCGCCCC); ATCAATCA, the known binding site for the human proto-oncogene Pbx-1 [41]. A similar site, ATCAATTA, has been shown to be bound *in vitro *by the *Drosophila *homolog of Pbx-1, the extradenticle (exd) protein [42]. Moreover, CEH-20C was identified as the *C. elegans *homolog of both Pbx-1 and exd. Other known sites discovered by FastCompare include CAGGTGA, similar to the known binding site for the Snail protein, a transcription factor involved in dorso-ventral pattern formation in *Drosophila *(published consensus [AG][AT][AG]ACAGGTG[CT]AC [43]), and TTCGCGC, the known binding site for the E2F proteins, a family of transcription factors involved in regulating the cell cycle in *Drosophila *and mammals (published consensus TTTCGCGC [44]). An E2F homolog has been identified in *C. elegans *and recently shown to be involved in cell-cycle regulation [45,46].

#### Position and orientation biases

**Figure 6:**
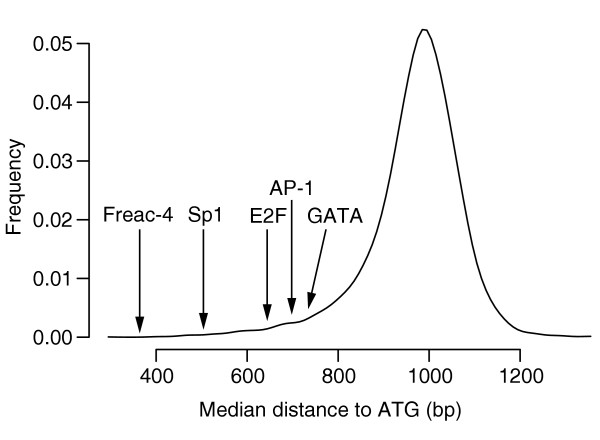
Distribution of median distances to ATG of all 7-mers, obtained when applying FastCompare to *C. elegans *and *C. briggsae*. For each 7-mer, a median distance to ATG was calculated using the positions of matches upstream of *C. elegans *genes within the conserved set for this 7-mer. The 8,170 median distances were then binned into 20-bp bins, and the resulting histogram was smoothed using a normal kernel. The median distances for several known binding sites in *C. elegans *are also indicated.

As in yeast, several of the known binding sites in *C. elegans *appear to be constrained in terms of position. Using the distribution of median distances for all 7-mers (see Materials and methods), we found *d*_0.025 _= 690 and *d*_0.975 _= 1,135. Among the 437 highest-scoring *k*-mers, we found that 75 are located below the lower threshold, a proportion that is much higher than the expected 2.5% (*p *< 10^-38^). The binding sites for forkhead-related activator-4 (Freac-4), Sp1, E2F and AP-1 are particularly constrained (see Figure 6). We found only 21 *k*-mers to be located further away from the distant *d*_0.975 _threshold. Interestingly, the most conserved *k*-mer among these 21, CCACCAGGA (rank 96), is found in the upstream regions of over- or underexpressed genes in 57 microarray conditions.

Note that for a few predicted elements (for example, CAGGTGA, rank 111), the median distance falls outside of the optimal window; this is due to the fact that, for these elements, the median distance does not correspond to the peak of the distribution of distances to ATG. Hence, for these elements, the optimal window provides a better descriptor of the positional bias than the median distance. Additional analysis reveals that several of the known binding sites discovered in this study are constrained in term of orientation. For example, the binding site for the GATA-factor(s) (as shown in Table 2) is significantly more often found in the 3' to 5' orientation, relative to downstream genes. Probably the most interesting finding is that the GAGA repeats appear to be strongly oriented 3' to 5' relative to their downstream genes. Indeed, 2,375 out of 3,557 (67%) of the AGAGAGA sites are oriented 3' to 5', a proportion that is much larger than the expected 50% (p < 10^-90^). This bias is confirmed by the fact that TCTCTCT alone (not taking into account its reverse complement) has a much higher conservation score (129.2) than AGAGAGA (34.3). We also found that several related motifs display a similar, albeit weaker, orientation bias, for example, GAAGAAG (*p *< 10^-16^), GGAGGAG (*p *< 10^-10^). It is interesting that all the GAGA repeats found to be necessary for correct expression of the *ceh-24 *and *unc-54 *genes are in fact TCTC repeats [22,23]. The conserved sets for TCTCTCT or AGAGAGA were not found to be enriched in any GO category. Note that this orientation bias is not due to genes with the repeats in their upstream regions being predominantly located on one strand, as these genes are approximately identically distributed on each strand (1,065/1,122, *p *= 0.89). Interestingly, conserved GAGA repeats in *D. melanogaster *were also found to be constrained in terms of orientation, but at a much lower significance (p < 10^-4^, see below). Although it is possible that the TCTC repeats are bound at the 5' untranslated region (UTR) mRNA level, the positional distribution of the conserved AGAGAGA sites does not indicate a strong positional bias with respect to ATG (D_ATG _= 893).

#### Novel predicted regulatory elements

FastCompare also returned many novel motifs; some of the most interesting ones are shown in Table 3. The top-scoring motif, CTGCGTCT, belongs to this category. A larger version of that motif, TCTGCGTCTCT, was found in a recent study to be necessary for the expression of several ethanol-response genes [47]. However, the very high conservation of this site suggests a broader role. It is interesting to note that this site was not significantly found upstream of under- or overexpressed genes in any microarray conditions (including the data from [47]). Interestingly, the most conserved *k*-mer found in yeast, the binding site for the Reb1 protein, had the same property. Moreover, this site displays a relatively strong orientation bias 5' to 3' (*p *< 10^-10^).

Several of the other novel predicted regulatory elements in Table 3 have interesting properties. For example, the fourth most-conserved *k*-mer, CGACACTCC, is one of the closest motifs to ATG, with a median distance of 234 bp, and its conserved set is strongly enriched in genes involved in positive regulation of growth (a biological process defined in GO as the increase in size or mass of all or part of the worm) (p < 10^-7^). Another predicted regulatory element, CGAGACC (rank 20), is found upstream of downregulated genes in 23 microarray conditions. Interestingly, it is found upstream of downregulated genes in a study measuring gene-expression changes at several time points during worm aging [48], in two distinct strains (*fer-15 *and *spe-9;fer-15*) and at similar time points (6, 9 and 10 days for *fer-15*, 9 and 11 for *spe-9;fer-15*). In addition, the functional enrichment of its conserved set points at a potential role in embryonic development (*p *< 10^-7^). Another strongly conserved and novel motif, CTCCGCCC (rank 14), was independently found upstream of almost all transcribed worm microRNA genes in a recent study [49].

#### Motif interactions

We found many interactions between the most conserved *k*-mers found at the previous stage. For example, the most conserved *k*-mer, TCTGCGTCT, is very often co-conserved with AGAGAGA. The high-scoring interaction between the DRE-like motif, AATCGAT and the putative E2F-binding site, TTTTCGC, also appears interesting. Indeed, the conserved sets for both *k*-mers are separately enriched significantly with genes involved in embryonic development, according to GO (*p *< 10^-8 ^and *p *< 10^-7^, respectively). However, the conserved set of genes having both elements in their upstream regions is even more enriched in this GO category (*p *< 10^-9^). TTTTCGC also seems to interact with the novel site CGACACTCC, and the corresponding conserved set is enriched with genes involved in modification-dependent protein catabolism (*p *< 10^-5^). The full list of motif interactions is available at [9].

### Flies

We applied FastCompare to the genomes of *D. melanogaster *and *D. pseudoobscura*, two species of *Drosophila *that diverged about 46 million years ago [50]. The number of orthologous ORFs between these two species is 11,306 and here we only consider 2,000-bp upstream regions. Using 5,000 bp instead produced similar results, but also produced additional putative binding sites (results are available at [9]). It takes approximately 10 minutes for FastCompare to process the corresponding 45 Mbp of sequences and calculate a conservation score for all 7-mers, 8-mers and 9-mers on a typical desktop PC.

#### Validations

**Figure 7:**
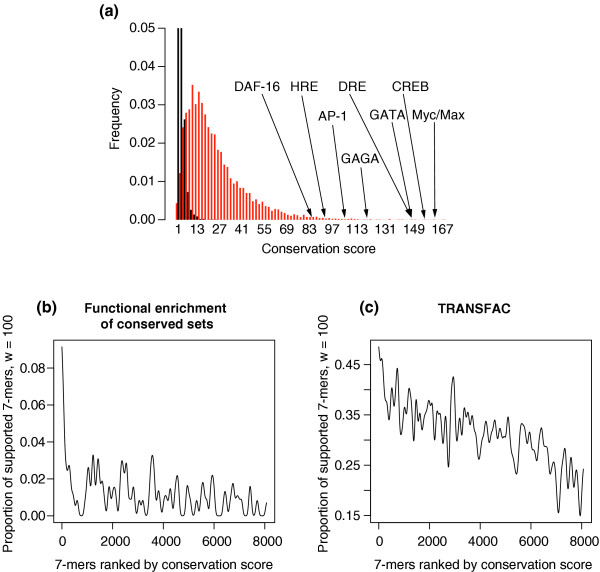
Validation of the conservation scores obtained when applying FastCompare to *D. melanogaster *and *D. pseudoobscura*. **(a) **Distributions of conservation scores for actual (red) and randomized (black) data, showing that high conservation scores are unlikely to be obtained from randomized data. Conservation scores for certain known regulatory elements are also indicated. Both distributions were constructed using bin sizes of 5, and the top portion of the figure is not shown for the purpose of presentation. **(b, c) **Proportion of 7-mers supported by different types of independent biological data (using windows of size 100, see Materials and methods) as a function of the conservation score rank, obtained when applying FastCompare to *D. melanogaster *and *D. pseudoobscura*. (b, c) strongly indicate that the frequency of support increases with conservation score as calculated by FastCompare.

The distribution of conservation scores shown in Figure 7a, for actual and randomized data, shows once again that the high conservation scores obtained with the real sequences are very unlikely to be achieved by chance. Also, as shown in Figure 7a, many known regulatory elements fall on the tail of the distribution.

As for the yeast and worm genomes, we used functional annotations (GO), expression data and known TRANSFAC sites to evaluate the FastCompare predictions. Unfortunately, expression data is often available for only a subset of genes and its analysis led to very few validations. However, Figure 7b,c clearly shows that functional enrichment of the conserved sets and TRANSFAC matches strongly correlate with conservation score. As with yeasts and worms, we focused on the 400 highest-scoring 7-mers, which are particularly well supported by the functional enrichment analysis (see Figure 7b). The simple processing described in Materials and methods yielded 469 *k*-mers (*k *= 7, 8 or 9), which we further analyze below.

#### Known regulatory elements

As shown in Table 4a, we found at least 16 distinct known regulatory elements among the 469 highest-scoring *k*-mers. The most conserved element, AACAGCTG, is similar to the site known to be bound by AP-4 (mammals) and MyoD (worms, flies and mammals). One of the most interesting predictions is TATCGATA (rank 12); this palindromic motif, known as the DNA replication-related element (DRE), has been experimentally proved to be necessary for proper expression of several cell proliferation-related genes in *D. melanogaster *[51] and, more recently, the genes encoding the TATA-binding protein (TBP) [52] and catalase [53] in the same organism. Interestingly, it is both the motif with the closest median distance to ATG (D_ATG _= 168), and the most over-represented *k*-mer (among the 469 highest scoring ones) within *D. melanogaster *upstream regions compared to exons, with a ratio of 5.39.

Several of the other predicted sites are known to be bound by *Drosophila *transcription factors involved in development. For example, FastCompare predicts TTTATGGC (rank 14) and TAATTGA (rank 24), the binding sites for two homeodomain transcription factors. The first site matches the TRANSFAC consensus binding site for Abd-B ([CG]NTTTATGGC), while the second site is the known consensus binding site for the Antennapedia (Antp) class of homeodomain proteins [54] (TAATTGA matches the TRANSFAC consensus binding site for Ubx, a member of the Antp class). FastCompare also predicts ATTTATGC, a site matching the TRANSFAC consensus binding site for the chicken CdxA protein ([AC]TTTAT[AG]), the homolog of the Caudal protein in *D. melanogaster*. Also, FastCompare predicts CAGGTGC, the binding site for the Snail repressor/activator protein, a transcription factor required for proper mesodermal development [43].

FastCompare also predicts ATTTGCATA (rank 3) as one of the most conserved putative regulatory elements between the two flies. This site is the binding site for the POU-domain family of transcription factors, and it is probably bound by one or several of the three POU-domain transcription factors in *Drosophila*: DFR, PDM-1 and PDM-2. These three proteins are involved in different stages of *Drosophila *development: DFR is expressed in midline glia and in tracheal cells [55], whereas the redundant PDM-1 and PDM-2 are essential for proper neuronal development [56].

Many of the known motifs found when comparing the two *Drosophila *genomes were also found when analyzing the worm genomes. For example, GAGA repeats are found to be strongly conserved, slightly oriented 3' to 5' (*p *< 10^-4^), and very significantly found upstream of genes involved in morphogenesis (*p *< 10^-23^). GTAAACA (rank 147), the DAF16-binding site in *C. elegans*, is also one of the most conserved sites between the two *Drosophila *genomes. This site is probably bound by dFOXO, the unique homolog of the *C. elegans *DAF16 protein in *D. melanogaster *[57].

As for both previous phylogenetic groups (yeasts and worms), the median distances to ATG for the conserved elements show that some of the predicted regulatory elements are severely constrained in terms of position. Among the most constrained *k*-mers are the DRE site (TATCGATA, D_ATG _= 168) and the known AP-4/MyoD binding site (AACAGCTG, D_ATG _= 373). However, both the optimal windows and the median distances in Table 4a show that, compared to previously studied organisms, a smaller number of conserved regulatory element are constrained. Using the distribution of median distances for all 7-mers, we find that the *d*_0.025 _= 798 and *d*_0.975 _= 1,126. Among the 469 highest scoring *k*-mers, 45 fall below 798 (*p *< 10^-13^) and 36 above 1,126 (*p *< 10^-8^), once again suggesting weaker positional constraints than in yeasts and worms, at least when considering the first 2,000 bp of 5' upstream sequences.

#### Novel predicted regulatory elements

FastCompare predicts many putative regulatory elements in *Drosophila *that to the best of our knowledge are unknown (Table 4b). One of these novel sites, CAGGTAG (rank 143), was found upstream of several genes that are activated before widespread activation of zygotic transcription (which begins during the 14th nuclear cycle), in several *Drosophila *species [58]; it was also found to be necessary for the early expression of several of these genes (*Sxl *and *sisterlessB*) in a subsequent study (J.R. ten Bosch, J.A. Benavides and T.W. Cline, personal communication). It is interesting to see that this particular site is significantly conserved upstream of genes involved in cell fate commitment (*p *< 10^-8^).

Some of these sites, such as the palindromic TTAATTA (rank 31), are found much more often in upstream regions than in exons (with an over-representation ratio of 3.07). Others, such as ACACACAC, are found to be significantly enriched upstream of genes in known functional categories (embryonic development, *p *< 10^-9^). The same site appears to be strongly oriented 5' to 3' (*p *< 10^-12^). Others, such as GTGTGACC or AAATGGCG, appear to be located closer to ATG than most other sites (D_ATG _= 296 and 592, respectively).

#### Motif interactions

We found many potential interactions between the most conserved sites discovered by FastCompare. For example, the POU-domain-binding site ATTTGCATA was found to be strongly co-conserved with TAATTGA, the Antp-binding site, and with many other potential homeodomain sites, such as AATAAAT and TAATTAA. The CACA repeats were also found to be co-conserved with several different sites, and in some cases, the set of genes having both sites simultaneously conserved in their upstream regions (conserved sets) was found to be enriched in certain functional categories, for example, ACACACAC and GAGAGAG, regulation of transcription (*p *< 10^-12^); ACACACAC and TAATTGC (an Antp variant site), embryonic development (*p *< 10^-5^). The full list of interactions is available at [9].

### Mammals

The much larger noncoding regions of mammalian genomes present significant challenges for computational motif discovery. Also, many repeat elements (for example, *Alu*) have colonized mammalian genomes and are likely to be conserved between closely related genomes. The distance between enhancers and the transcriptional start of the genes they regulate can be extremely large, reaching tens of kilobases. Finally, gene predictions and gene boundaries are still largely unverified experimentally for a large number of genes.

We applied FastCompare to the genomes of *H. sapiens *and *M. musculus*,, which diverged about 75 million years ago [59]. The number of orthologous ORFs between these two species is 15,983 and again, we have only considered 2,000-bp upstream regions. As in flies, using 5,000-bp instead produced similar results. It takes approximately 15 minutes for FastCompare to process the corresponding 60 Mbp of sequences and calculate a conservation score for all 7-mers, 8-mers and 9-mers on a typical desktop PC.

#### Validations

**Figure 8:**
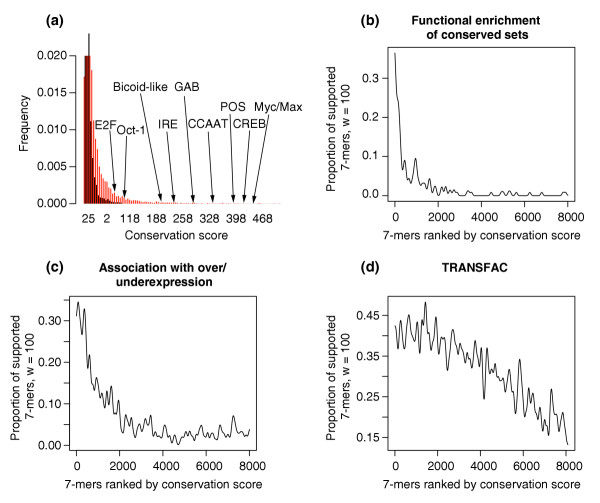
Validation of the conservation scores obtained when applying FastCompare to *H. sapiens *and *M. musculus*. **(a) **Distributions of conservation scores for actual and randomized data, showing that high conservation scores are unlikely to be obtained by chance. Conservation scores for some known regulatory elements are also indicated. Both distributions were constructed using bin sizes of 5, and the top portion of the figure is not shown for the purpose of presentation. **(b-d) **Proportion of 7-mers supported by different types of independent biological data (using windows of size 100, see Materials and methods) as a function of the conservation score rank, obtained when applying FastCompare to *H. sapiens *and *M. musculus*. (b-d) strongly indicate that the frequency of support increases with conservation score as calculated by FastCompare.

Unlike the other genomes considered so far, the output of FastCompare from the mammalian genomes is dominated by GC-rich sequences, probably corresponding to CpG islands (GC-rich regions known to be associated with the promoters of many genes). However, analysis of the FastCompare output yielded the same validations as for other species. Indeed, the distribution of conservation scores obtained on actual and randomized sequences shows that high conservation scores are very unlikely to be obtained by chance (Figure 8a). As with other species, many known regulatory elements are on the tail of the distribution (Figure 8a). Also, as shown in Figure 8b-d, more *k*-mers are found upstream of over or underexpressed genes, more *k*-mers have their conserved set enriched with GO functional categories, and more *k*-mers match TRANSFAC consensus sites as the conservation score increases.

We found that masking *Alu *repeats did not influence the output of FastCompare (data not shown). To overcome the overabundance of GC-rich sequences in the FastCompare output, we use longer *k*-mers as starting points, namely 8-mers instead of 7-mers. We started with the 600 highest-scoring 8-mers, and replaced each of these 8-mers by one of its substrings (7-mer) or one of its superstrings (9-mer), when their conservation score is higher. We then removed duplicates in the list and added the high-scoring 9-mers that have no substrings within the list. This procedure yielded 284 *k*-mers (*k *= 7, 8, 9). Subsequent validation was limited to this small set of high-scoring predictions.

#### Known regulatory elements

As shown in Table 5a, we found 17 distinct known regulatory elements among the 284 highest-scoring *k*-mers. Among these are the well characterized sites for the Sp1, C/EBP, CREB and Myc/Max proteins or families of proteins. These four sites reside very close to ATG (their median distance to ATG is between 100 and 250 bp), suggesting that the four proteins (or families of proteins) may be involved in intimate interactions with the transcriptional complex. Sp1 is an ubiquitous transcription factor, involved in the basal expression of a large number of genes in mammals (see [60] for review). The CCAAT/enhancer binding protein (C/EBP) has been implicated in the regulation of cell-specific gene expression mainly in hepatocytes, adipocytes and hematopoietic cells (see [61] for review). Both Sp1 and C/EBP are constitutive transcription factors whose presence is necessary for significant induction of a large number of genes [62]. The CRE-binding protein (CREB or CBP) is a transcription factor that binds cyclic AMP (cAMP) response elements (CREs) in the promoters of specific genes, and functions as a co-activator for a large number of other transcription factors (see [63] for review). The Myc/Max heterodimer binds the CACGTG sequence, and also acts as a transcriptional activator (see [64] for review).

Interestingly, we found that some of the most conserved interactions between *k*-mers (see Materials and methods) involve Sp1-binding sites (CCCGCCC or CCGCCCC) with other known sites such as CACGTGAC (Myc/Max), TGACGTCA (CREB), CGCAGGCGC (unknown), GCCAATC (CCAAT-box) and ACTTCCG (Ets), and that the median distances between these sites are relatively small (138, 164, 200.5, 234 and 234, respectively).

Among the other predicted regulatory elements returned by FastCompare are CCGCCTC, a site known as the insulin response element [65]; CGGAAGTGA, a site known to be bound by the GA-binding protein in human [66]; CGCATGCG, a site known as the palindromic octamer sequence, which was found at 132 bp (relative to ATG) upstream of the inosine-5'-monophosphate dehydrogenase type II gene in human, and shown to be functional in resting and activated T cells using site-directed mutagenesis, *in vivo *footprinting and electrophoretic mobility shift assay (EMSA) [67]; TTTCGCGC, the E2F-binding site; TAATCCCAG, a site known to be bound in *D. melanogaster *by the anterior morphogen Bicoid, and also recently shown to be bound in human by *Goosecoid-like *(GSCL) [68]. Interestingly, this site has a relatively strong orientation bias 3' to 5' (*p *< 10^-14^). It is also the site with the strongest over-representation in upstream regions compared to exons that we observed, with a ratio of 7.06.

FastCompare also predicts ATTTGCAT, the binding site for the POU-domain Oct-1 and Oct-2 proteins, known to bind the promoter and intronic enhancer of immunoglobulin genes [69]; it also returns GGAAGTCCC, a site that was shown to bind NFκB [70,71], a transcription factor involved in a variety of pathways (including inflammation, response to infection and oxidative stress, and apoptosis).

**Figure 9:**
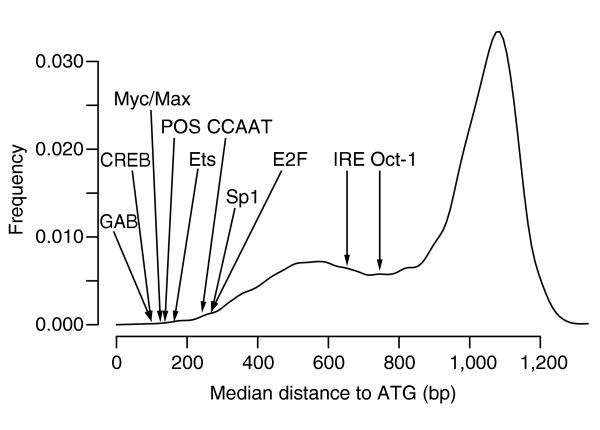
Distribution of median distances to ATG of all 7-mers, obtained when applying FastCompare to *H. sapiens *and *M. musculus*. For each 7-mer, a median distance to ATG was calculated using the positions of matches upstream of *H. sapiens *genes within the conserved set for this 7-mer. The 8,170 median distances were then binned into 20-bp bins, and the resulting histogram was smoothed using a normal kernel. The median distances for several known binding sites in *H. sapiens *are also indicated.

The distribution of distances to ATG for all 7-mers (Figure 9) shows an interesting bimodal shape, indicating that a large number of short sequences are constrained to reside around 500 bp to ATG. We calculated *d*_0.025 _= 342 and *d*_0.975 _= 1,185 and found that 83 *k*-mers among the 284 highest-scoring ones have a shorter median distance than 342 (*p *< 10^-63^) and only 11 have a larger median distance than 1,185. Indeed, a majority of the known sites identified by FastCompare are preferentially located near the 5' start of genes, with some elements being very close to ATG (for example, the CREB site, whose median distance to ATG is 107, whereas the optimal window is [0;1,000]). Nonetheless, a few known motifs do not seem to show any positional constraints. For example, the Bicoid-like site TAATCCCAG has a median distance to ATG of 1,258.

#### Novel predicted regulatory elements

FastCompare identifies many putative regulatory elements which to the best of our knowledge are novel (Table 5b). Some of these predicted regulatory elements are found upstream of over- or underexpressed genes in many microarray conditions. One example is CCCCAGC, which is significantly found upstream of overexpressed genes in 21 conditions (out of 30) of the human cell-cycle experiment [72]. Other conserved elements are found much more often in upstream regions than in exons, for example, CCCCTCCC or TCTCGCGA, with ratios of 5.12 and 4.45, respectively. Others appear to be positionally constrained, for example, the palindromic CTGCGCA with an optimal window [0;300] and a median distance to ATG of 199, or constrained in terms of orientation, for example, GTGAGCCAC, which is significantly oriented 5' to 3' (*p *< 10^-6^).

### Inter-groups comparisons

To gain a better understanding of the network-level conservation of regulatory elements between the different phylogenetic groups, we compared the results we obtained by applying FastCompare to yeasts, worms, flies and mammals in the previous sections. We calculated the overlap (and its significance) of the 400 highest-scoring 7-mers and 8-mers found for each phylogenetic group. As shown in Table 6a,b, the number of shared predicted sites correlates with phylogenetic distance (the number of high-scoring putative motifs that two phylogenetic groups have in common decreases as the phylogenetic distance between the groups increases). All of the overlaps were found to be statistically significant, except for the yeast-human comparison. For both 7-mers and 8-mers, the best overlap is the one obtained between the two invertebrate phylogenetic groups: worms and flies. Indeed, simple observation of the identified known regulatory elements in Tables 2 and 4a reveals that these two organisms have a large number of predicted binding sites in common.

However, when we looked at the overlap between conserved sets for identical high-scoring *k*-mers in different phylogenetic groups (after determination of reciprocal best BLAST hits between the considered species), we found little overlap. The only significant overlap we found (after Bonferroni correction) was between the GATA sites (GATAAGA) in worm and fly (*p *= 2.5 × 10^-4^). As a control, we performed the same analysis within the yeast phylogenetic group, using the *S. cerevisiae*/*S. bayanus *and *S. paradoxus*/*S. mikatae *400 most conserved 7-mers. One hundred and ninety-five sites were found in both groups of 7-mers, and for all of them, the overlaps between the conserved sets obtained separately in the *S. cerevisiae*/*S. bayanus *and *S. paradoxus*/*S. mikatae *analyses were highly significant, with hypergeometric *p*-values < 10^-40^. Therefore, our results strongly suggest that, while transcription factors have largely retained their ability to recognize specific DNA sites, their targets have largely changed through appearance or disappearance of those binding sites in promoters. This hypothesis is supported by recent analysis of the fission yeast cell cycle using microarrays, which showed that the role and the binding sites for several of the main transcription factors involved in regulating the yeast cell cycle (Swi4/Mbp1, Fkh1/Fkh2, Swi5/Ace2) are conserved between budding and fission yeasts (which diverged about 1 billion years ago), but the sets of genes that they regulate overlap much less than expected (only about 50 orthologous genes are cell-cycle-regulated in both species) [73].

It is particularly interesting to consider the seven 8-mers that are top predictions for all three multicellular phylogenetic groups (note that many more 7-mers are conserved between these groups). These sites include the CRE (TGACGTCA, GACGTCAC and ATGACGTC), the POU-domain binding site (ATTTGCAT), and the HRE (CAAGGTCA). A fourth site is also shared (GCCACGCC, CCACGCCC), which to the best of our knowledge is a novel motif. Its strong over-representation in upstream regions compared to coding regions, and its closeness to ATG (median D_ATG _= 230 for GCCACGCC) make it a promising candidate for experimental testing. Interestingly, the location constraints on these conserved sites can vary across phylogenetic groups. For example, the CRE appears weakly constrained in worms and flies in terms of distance to ATG (D_ATG _= 708 and 825, respectively), but is very close to ATG in mammalian genomes (D_ATG _= 107). However, the distances to ATG of the POU-domain-binding sites (862, 882 and 729, respectively) indicate that their positional constraints are shared among the phylogenetic groups. The same holds for the HRE binding site (845, 1,015.5 and 895, respectively).

## Discussion and conclusions

We have presented a powerful approach for discovering transcriptional regulatory elements that are globally conserved between pairs of genomes. Our approach requires only two unaligned genomes, thus allowing the use of genomes of arbitrary divergence and those with extensive rearrangements of noncoding regions. Moreover, our motif-finding strategy does not use any parameters other than a conservation score threshold, used to separate presumptive functional from nonfunctional motifs. We have shown that such thresholds can be roughly estimated using independent biological data, when available. Our approach is also computationally efficient: whole eukaryotic genomes can be processed in minutes on a typical computer. In turn, this efficiency allows FastCompare to explore exhaustive pattern lists.

Our results show that FastCompare can recover most of the known functional binding sites in *S. cerevisiae *when its upstream regions are compared to those of a related species, *S. bayanus*. We comprehensively explored the globally conserved motif content between worms, flies and mammalian genomes, discovering large sets of known and novel motifs. The use of external information (expression data, functional categories, TRANSFAC, chromatin IP and known motifs) clearly shows that our method is able to detect conserved and functional motifs in all the phylogenetic groups that we studied. In all analyses, we have shown that some of the discovered known or novel motifs were severely constrained, either in terms of position relative to the start of translation or in orientation. We also observed that some of the known or novel motifs are co-conserved within upstream regions, potentially revealing interactions between the (often unknown) transcription factors that bind them.

**Figure 10:**
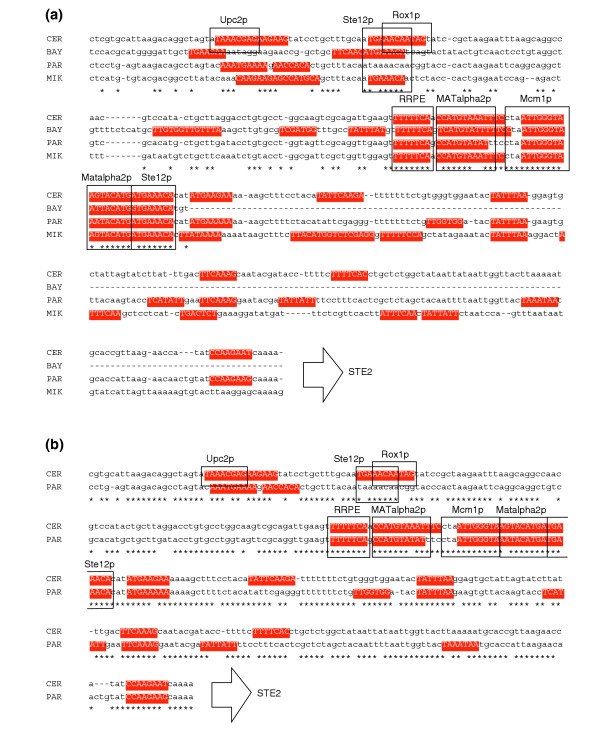
Partial representation (most proximal region) of the aligned 1 kb upstream regions of the *S. cerevisiae STE12 *gene and its orthologs. **(a) **The highest scoring 7-mers found by FastCompare in a comparison between *S. cerevisiae *and *S. bayanus *are highlighted. FastCompare correctly predicts the conserved and experimentally verified binding sites for Mcm1, Matalpha2 and Ste12 (proximal) (see [8] for review). A more distal non-verified binding site for Ste12, and a RRPE site close to the distal Matalpha2 are conserved between the four species, and also predicted by FastCompare. FastCompare predicts several nonconserved sites in each species. For example, in *S. cerevisiae*, it identifies a Rox1-binding site overlapping with the second Ste12 site, and a putative Upc2-binding site. **(b) **Aligned 1 kb upstream region of the *S. cerevisiae *STE2 gene and its *S. paradoxus *ortholog only, with the same highlighted 7-mers as in (a). Since the two yeast species diverged very recently, the two upstream regions appear highly conserved. However, using the FastCompare output allows efficient selection of verified and putative binding sites. CER, *S. cerevisiae*; Bay, *S. bayanus*; Par, *S. paradoxus*; Mik, *S. mikatae*.

We have created a set of web tools to superimpose the most globally conserved *k*-mers discovered by FastCompare to user-supplied sequences or multiple alignments. An example is shown in Figure 10a, in which the upstream regions of the *STE2 *gene (encoding the alpha-factor pheromone receptor) from four different yeast species were aligned using ClustalW, and the most globally conserved *k*-mers are highlighted. All experimentally determined sites for *STE2 *were also predicted to be globally conserved by FastCompare. Moreover, several other sites also appear to be conserved, both at the global level (predicted by FastCompare) and the local level (shown by the multiple alignment). In Figure 10b, the same analysis was performed on only two orthologous upstream regions instead of four. Many more sites appear to be locally conserved than when using four species, but the globally conserved sites found by FastCompare allow the efficient selection of experimentally verified and putative binding sites. These tools should be particularly useful in designing stepwise promoter deletions and site-directed mutagenesis experiments for understanding the regulatory code of specific genes.

While powerful, our approach has potential limitations. Our current approach allows matches to a given *k*-mer to be on different strands within pairs of orthlogous upstream regions. This flexibility substantially increases the number of *k*-mers that are supported by independent biological data (that is, true positives), at least for yeasts and worms (data not shown). However, it is difficult to evaluate whether this flexibility introduces more true positives than false positives. Also, transcription factors often bind several slightly distinct sites with different affinities, and it is widely acknowledged that binding-site degeneracy is better captured by using position-weight matrices (PWM) instead of *k*-mers or consensus patterns [74]. To evaluate whether weight matrices would display better conservation scores, we calculated a conservation score for weight matrices corresponding to 20 well characterized yeast binding sites, and compared them to the conservation scores obtained for the best *k*-mers that unambiguously correspond to the same binding sites. Conservation scores for weight matrices were calculated as described for *k*-mers in Materials and methods, except that we used the weight-matrix score thresholds that maximize the significance of the overlap between the two sets of ORFs containing matches to the weight matrices in each species. This involves progressively lowering the score threshold by small increments, and for each threshold, calculating the overlap and its hypergeometric *p*-value. We then choose the score threshold corresponding to the most significant *p*-value, and use the negative natural logarithm of this *p*-value as the conservation score. As shown in Table 7, only in 11 cases out of 20 did weight matrices have a higher conservation score than the corresponding *k*-mers. These results suggest that *k*-mers provide results that are almost as good as those obtained using weight matrices, when utilizing the network-level conservation criterion. One reason why, in many cases, *k*-mers have a higher conservation score than weight matrices may have to do with the more narrow selection of *k*-mers for binding sites with similar or identical affinities. In fact, we recently showed that PWM scores, widely seen as proxies for binding affinity, are statistically conserved in a comparison between *S. cerevisiae *and *S. bayanus *[6]. In the context of the present study, the different *k*-mers representing each transcription factor binding site may be defining affinity classes that are more strongly conserved than a looser definition of a binding site represented by a weight matrix. Recent work in bacteria has established the importance of binding affinity, especially with respect to coordinating the temporal order of events [75].

However, Table 7 shows that the conservation score for weight matrices describing very degenerate binding sites, such as RAP1, is significantly higher than the conservation score obtained for the best corresponding *k*-mer. This suggest that our *k*-mer based approach is limited in its ability to discover highly degenerate binding sites.

As shown by our inter-group analysis, many regulatory elements have remained functional across evolution, but few have remained upstream of the same genes. The network-level conservation principle thus appears less applicable to species that diverged very long ago. For example, when we compared the *Drosophila *and mosquito genomes (which diverged approximately 400 million years ago), we only found a handful of *k*-mers (interestingly including GATA-factor and Myc/Max binding sites) to have conservation scores above those obtained from randomized data.

There are also several directions in which our approach could be extended. From a methodological standpoint, the approach could be extended to take into account local over-representation of identical or nearly identical copies of the same binding sites, a well known feature in the promoter regions of higher eukaryotic species [16]. To discover highly degenerate regulatory elements, *k*-mers could be used to seed weight matrices whose individual weights could be optimized for network-level conservation, using stochastic optimization procedures (for example, simulated annealing; Mike Beer, personal communication). Introns and downstream noncoding regions could also be explored using our approach, as these regions are known to harbor functional regulatory elements in metazoan genomes. While our approach can deal with genomes presenting arbitrary levels of divergence and rearrangements, it would be interesting to investigate how global alignments or suboptimal and non-overlapping local alignments [76] could be used to filter out regions of non-conservation. This approach would be particularly interesting when analyzing very long upstream regions, in order to increase the signal-to-noise ratio. Finally, mRNA 3' UTRs could be compared in order to find specific downstream regulatory elements involved in post-transcriptional mRNA regulation (for example, mRNA localization, decay or translational repression).

## Materials and methods

### Outline of approach

First we determined orthology relationships between ORFs on the basis of reciprocal best BLAST hits (Figure 1a) and extracted the corresponding upstream regions from the genome sequences. Then, we considered every possible short DNA sequence of length *k *(*k*-mer, with *k *between 7 and 9) as a candidate regulatory element. For each *k*-mer, we found the set of ORFs whose upstream regions contain at least one exact match to the *k*-mer, anywhere in the upstream region, in the first genome. We did the same for the second genome, obtaining another set of ORFs. Then, we calculated the overlap between the two sets and assessed its statistical significance (Figure 1b). The statistical significance of the overlap provides a measure of conservation with which we score and rank every possible *k*-mer (Figure 1c). Note that our approach is very different from the classical *k*-tuple DNA sequence-analysis methods [77,78], which are not based on comparative genomics and are local methods; that is, they only deal with single promoters or small sets of functionally related promoters (while our approach provides a genome-level measure of conservation for candidate regulatory elements).

### Sequence sources and orthology determination

Sequence data were downloaded from the *Saccharomyces *Genome Database (SGD) for all yeast species considered in this paper; worm (*C. elegans *and *C. briggsae*), *Drosophila *(*D. melanogaster*), human (*H. sapiens*) and mouse (*M. musculus*) sequence data were downloaded from Ensembl [79]. The *D. pseudoobscura *genome sequences (contigs) were downloaded from [80]. The upstream regions used in this study are immediately adjacent to the ATG codons of their downstream genes, and are 1-kb long (yeasts) or 2-kb long (worms, flies and mammals). Note that transcription-factor-binding sites generally reside in the region situated upstream of the transcription start site. Unfortunately, not all genes have well annotated transcription start sites. This problem should not, however, strongly influence the output of FastCompare, as distances between start of transcription and start of translation should be at most on the order of a few hundred base-pairs (except in certain cases, for example when 5' UTRs are interrupted by long introns). However, as gene structures become better annotated (mainly as a result of massive cDNA sequencing projects) and promoter regions become more accurately delimited, we expect that the ability of FastCompare to discover regulatory elements will be significantly improved.

Orthology information provided by Ensembl or by Kellis *et al*. [4] was used throughout this study, when available. Ensembl provides strong homology relationships between genes from different species, but does not provide reciprocal best matches. Therefore, we determine reciprocal best matches using the provided sequence identity between homologous genes. When orthology information is not available in Ensembl (for example, between *D. melanogaster *and *D. pseudoobscura*, or between distant species such as *S. cerevisiae *and *C. elegans*), we determine orthologs using the reciprocal best BLAST hits approach.

### Motif-finding algorithm and simple clustering

Given a value of *k*, we first generated the set of all possible *k*-mers and removed half of them on the basis of reverse complementarity. We also removed *k*-mers with very low complexity and which are over-abundant in the intergenic regions of the genomes we analyzed (that is, those that contain *k *- 1 or more As or Ts), as these sequences are unlikely to be regulatory elements. Every remaining *k*-mer (that is, 8,170 for *k *= 7) is then considered as a candidate regulatory element. For each *k*-mer, we found the set of ORFs in the first species that have at least one exact occurrence of the *k*-mer in their upstream regions. We then found the set of ORFs in the second species that have at least one occurrence of the same *k*-mer in their upstream region. Importantly, the matches can be anywhere in the upstream regions: they do not have to be at the same positions in two orthologous upstream regions (as with multiple alignment) and can be on any strand. Since both functional and non-functional elements are expected to be conserved between two closely related species, the two sets are expected to overlap. However, under the network-level conservation principle, the extent of the overlap - and therefore its statistical significance - will be even greater for *k*-mers that represent functional transcription factor binding sites. The significance of the overlap can be measured using the hypergeometric distribution. The probability of two sets of size *s*_1 _and *s*_2_, drawn from a set of *N *elements, to have *i *or more elements in common is given by :



In this way, all *k*-mers can be ranked by their hypergeometric *p*-values. It is important to note that due to basal conservation (that is, conservation arising from common ancestry), the hypergeometric *p*-values will generally be very small for most *k*-mers. Therefore, we only use these *p*-values as relative measures of network-level conservation and focus on *k*-mers with the greatest conservation. For simplicity, we define the 'conservation score' to be the negative logarithm (base *e*) of the hypergeometric *p*-value obtained for a given *k*-mer. Therefore, the more extensive the overlap between the two sets, the higher the conservation score. Also, for the same *k*-mer, we call 'conserved set' the set of ORFs corresponding to the overlap between the two sets of orthologous ORFs containing at least one exact match to the *k*-mer in their upstream regions. Conserved sets are used throughout this study to get insights into the function of the most conserved *k*-mers, using functional annotation [81,82], chromatin IP [1], known motifs, and to evaluate whether these *k*-mers are constrained in terms of position or orientation.

The current FastCompare implementation handles *k*-mers with a user-specified gap (termed gapped *k*-mers), which is a straightforward extension of the approach described above. The conservation score returned by FastCompare is independent of the size of the patterns (that is, the value of *k*); therefore *k*-mers with different sizes, and gapped *k*-mers (for example, CGTNNNNNNTGA) can be compared.

We use the following strategy when applying FastCompare to pairs of genomes. First, we calculate conservation scores for all 7-mers, 8-mers and 9-mers. We then retain only the *m *highest-scoring 7-mers, with *m *chosen according to independent biological data (alternatively, *m *could be chosen according to the estimated number of transcription factors in the species being considered). We then replace each of the retained 7-mers by an 8-mer (if there is one) with higher conservation score for which the considered 7-mer is a substring. We also include within the final list the 8-mers which do not have any substrings within the *m *7-mers. We then repeat the same process for the retained 8-mers, replacing each of them by its higher scoring 9-mer superstring if there is one, and add the 9-mers that do not have any substring within the 8-mers. This strategy thus allows the optimal length for candidate regulatory elements to be determined.

FastCompare is implemented in the C language and uses efficient data structures (hash tables and prefix trees [83]). For a given value of *k*, the worst-case time complexity is *O*(*kn *+ 4^*k*^(*p *+ *k*)), where *n *is the total amount of upstream sequences and *p *is the total number of orthologous pairs. Note that the first term is generally much larger than the second one; therefore the complexity of our approach can be seen as linear in the combined sizes of the genomes to be compared (when *k *is restricted to 7, 8 and 9). The calculation of hypergeometric *p*-values involves factorials of large integers, so we use specialized C routines, as described in [84]. FastCompare runtimes provided in the Results section are obtained using a standard desktop PC (2.0 GHz CPU, 1 GB RAM).

### Discovering positional constraints for conserved regulatory elements

As described in Results, we applied FastCompare to 1 kb (yeast) or 2 kb upstream regions (worms, flies and mammals). While these lengths are reasonable, they are somewhat arbitrary, and it is known that some regulatory elements are constrained to be within specific distances (often shorter than 1 kb) from the start of transcription, reflecting mechanistic constraints for transcription factor-transcription factor or transcription factor-RNA polymerase interactions [11]. Moreover, some regulatory elements have orientation biases (see [11,12] for examples). To discover such constraints, we analyzed the most conserved *k*-mers found at the previous stage in the following ways.

First, for each high-scoring *k*-mer, we calculated the median distance to ATG (as the start of transcription is generally not known) for the set of all (non-overlapping) occurrences of this *k*-mer within the upstream regions of its conserved set (see previous section for a definition of the conserved set of a given *k*-mer). To statistically assess whether the median distance to ATG for a given *k*-mer is unusually small or large, we built the distribution *P*(*d*) of median distances to ATG, for the entire set of 8,170 7-mers. We first created a histogram by binning the median distances to ATG for all 7-mers into 20-bp bins, and then smoothed the histogram (using a normal kernel and a bandwidth of 50 as implemented in the *ksmooth *function of the R statistical software package). Then, using numerical integration, we sought the distance thresholds *d*_0.025 _and *d*_0.975 _such that *P*(*d *<*d*_0.025_) = 0.025 and *P*(*d *<*d*_0.975_) = 0.975. We then considered the median distance to ATG for a given *k*-mer as unusually short or long when it is less than *d*_0.025 _or greater than *d*_0.975_, respectively.

For each *k*-mer, we also sought the sequence window which maximizes the conservation score by progressively shortening all upstream regions (all having equal lengths) by 100 bp increments from the 5' end. Then, we did the same from the 3' end using the optimal 5' end found in the previous step. Evaluating every possible window whose length is a multiple of 100 bp almost always yields identical results. We then calculated the conserved sets for these windows, and output the orientation (strand) for each *k*-mer occurrence within its conserved set (palindromes were counted on both strand).

Finally, using the results of the previous step, for each *k*-mer, we used the binomial distribution to assess whether the proportion of occurrences of this *k*-mer (within its conserved sets) on one strand is significantly smaller (or larger) than 0.5. Binomial *p*-values less than 0.05 (after Bonferroni correction) are considered significant.

### Motif interactions

It is now known that the regulatory code governing the expression of genes is combinatorial [11,85,86]. The network-level conservation principle can be trivially extended to discover interactions (that is, co-conservation) between two *k*-mers. To focus on heterotypic interactions, we only examined *k*-mers that differ by more than *l *nucleotides, after optimal ungapped alignment. We tested several values of *l *and found that *l *= 4 was most appropriate when using 7-, 8- and 9-mers. Then, we proceeded as described above, except that instead of seeking two sets of ORFs (one for each species) whose upstream regions contain a single *k*-mer, we sought the two sets of ORFs that contain the two *k*-mers simultaneously. Once these two sets were available, we evaluated the extent of their overlap as described above, and rank interaction pairs according to their conservation score.

### Validations

We used randomized data to show that high conservation scores (obtained as described above) are unlikely to be obtained by chance, and independent biological information to assess the ability of FastCompare to predict functional regulatory elements by giving them a high conservation score. We also estimated the over-representation of predicted regulatory elements in upstream regions compared to coding regions.

#### Validation using randomized data

Our goal was to generate new pairs of upstream regions that are conserved at the same level of divergence as the actual sequence data. We align each pair of orthologous sequences using the Needleman-Wunsch algorithm [87], and calculate substitution frequencies between all pairs of nucleotides (A → A, A → T, and so on). Then, we reconstructed new pairs of orthologous sequences by mutating one of the sequences in each initial pair using the estimated frequencies. Generating the sequences to be mutated using locally estimated first-order Markov models yielded the same results.

#### Validation using independent biological information

The proportions of 7-mers supported by each type of independent data, as presented in Figures 3, 5, 7 and 8, is calculated as follows. In these figures, support for a given 7-mer is considered as binary, and depends on whether the 7-mer meets the particular validation criterion or not (or whether it is found upstream of over- or underexpressed genes, in at least one microarray condition, see below). 7-mers are first sorted by conservation score, and the proportion of supported 7-mers were calculated using a sliding window of 100 7-mers. For each window and each type of independent biological data, we simply calculated the number of 7-mers for which support is available and divided this number by 100.

##### Functional annotations

Yeast (*S. cerevisiae*), worm (*C. elegans*), fly (*D. melanogaster*) and human (*H. sapiens*) functional categories and corresponding ORF annotations were downloaded from the MIPS [88] and GO [89] websites. The statistical significance of the functional enrichments within sets of ORFs was evaluated using the hypergeometric distribution, as discussed above. Hypergeometric *p*-values for functional enrichment were not corrected for multiple testing, but only *p*-values smaller than 10^-4 ^are reported, providing a slightly less stringent thresholds than Bonferroni corrections.

##### Known motifs

Weight matrices corresponding to known yeast motifs were obtained from Gibbs sampling-based motif finding on chromatin IP data [1], functional categories and clusters of co-expressed genes [85]. Only high-confidence binding sites (that is, sites confirmed by several sources including the literature) were included in our list of known motifs. We label a given *k*-mer as a known motif if it meets the following two criteria. The first is significant overlap (*p *< 10^-4^) between the conserved set for the given *k*-mer and the set of ORFs whose upstream regions contain at least one match to the known motif (the sets of ORFs were defined using ScanACE with the weight matrix for the known motif, and with the standard average minus two standard deviations threshold [7]). The second criterion is strong sequence similarity between the considered *k*-mer and the known motif weight matrix. To evaluate this similarity, we turn the considered *k*-mer into a weight matrix of 0s and 1s, and use CompareACE [7] to calculate the Pearson correlation between the weights of this matrix and the weights of the known motif weight matrix; correlation coefficients > 0.65 are considered significant. Finally, for a given *k*-mer, we report the known motif for which the above hypergeometric p-value is the smallest.

##### *In vivo *binding data (chromatin IP)

Genome-wide binding locations were previously evaluated for 106 transcription factors in *S. cerevisiae *[1]. For each transcription factor, we retain the set of ORFs with *p*-value < 0.001 (see [1] for details of the error model). To evaluate a given *k*-mer with respect to chromatin IP, we evaluate the statistical significance of the overlap between the conserved set of the considered *k*-mer and the set of ORFs defined as described above corresponding to each transcription factor. We report the most significant chromatin IP enrichment, with hypergeometric *p*-value < 10^-4^.

##### TRANSFAC

The 309 weight matrices and corresponding consensus patterns for known transcription factor binding sites were downloaded from [90,91]. *k*-mers were then simply matched to the consensus patterns. We eliminated consensus patterns that match too many *k*-mers, by matching each of them to all (8,170) 7-mers and removing consensus patterns that matched more than 50 7-mers.

##### Microarray expression data

Expression data for all species considered were downloaded from diverse sources [92,93]. Overall, we downloaded 765 microarray conditions for *S. cerevisiae*, 555 conditions for *C. elegans*, 156 conditions for *D. melanogaster*, and 1,384 conditions for *H. sapiens*. We use these expression data in the following way.

We evaluated the over-representation of each *k*-mer in the upstream regions of genes that are themselves over- or underexpressed in certain microarray conditions. Over- or underexpressed genes are operationally defined as having a log ratio of intensity above average plus two standard deviations, or below average minus two standard deviations, respectively (averages and standard deviations are calculated for each condition; using fold changes instead of standard deviations produced roughly the same results). To evaluate the over-representation of a given *k*-mer in a given microarray condition, we defined as *O*_1 _the set of overexpressed genes in this condition, and as *O*_2 _the set of ORFs whose upstream regions contain at least one occurrence of the considered *k*-mer, genome-wide. Then, we evaluated the significance of the overlap between *O*_1 _and *O*_2 _using the hypergeometric distribution, as described above. Overlaps whose hypergeometric *p*-value is smaller than 0.05 (after Bonferroni correction) were considered significant. We proceeded separately with the set of underexpressed genes in the same way. The total number of microarray conditions (overexpressed plus underexpressed) for which a *k*-mer was found to be significantly over-represented is reported. Note that we do not use the conserved set for the considered *k*-mer here, as we do not want to restrict our analysis to orthologous genes. Indeed, except for yeast, microarrays often contain only a fraction of all genes within the considered organism. In these cases, the overlap between conserved sets and over- or underexpressed genes can be very small, reducing statistical power. Using all genes, therefore, increases our power to detect significant associations, while retaining a uniform approach for all species considered.

#### Over-representation in upstream regions compared to coding regions

As shown in [94] for the yeast RAP1 transcription factor, some transcription factors bind intergenic regions much more frequently than they bind coding regions. Consequently, it is expected that sequences corresponding to regulatory elements are more often present in intergenic regions than in coding regions. To evaluate this bias, we calculate the ratio of the number of genes that have the *k*-mer in their upstream regions over the number of genes that have the *k*-mer in their coding regions (using only exons), and we correct this ratio using the average length of the upstream and coding regions.

### Availability

The FastCompare implementation, all the sequences, and results are available on our website [9].

## Figures and Tables

**Table 1:** Known regulatory elements obtained when applying FastCompare to *S. cerevisiae *and *S. bayanus*

Name	Sequence	Rank	D_ATG_	W_ATG_	U/C	Motif	ChIP	Experiment	Best MIPS enrichment
Bas1	AAGAGTCA	159	307	[0;500]	1.24	BAS1	-	2(1/1)	Amino-acid metabolism (*p *< 10^-6^)
Cbf1	CACGTGA	3	368	-	2.70	CBF1	CBF1	6(3/3)	Amino-acid metabolism (*p *< 10^-6^)
Ecm22/Upc6	TAAACGA	59	362	[100;500]	1.36	-	-	11(9/2)	Lipid, fatty-acid and isoprenoid biosynthesis (*p *< 10^-8^)
Fkh1/2	TAAACAAA	88	353	-	1.73	FKH1	FKH2	2(1/1)	-
Gcn4	TGACTCA	160	323.5	[0;400]	1.02	GCN4	GCN4	102(76/26)	Amino acid biosynthesis (*p *< 10^-29^)
Gcr1	TGGAAGC	260	663	[600:1000]	1.24	GCR1	-	4(4/0)	-
Gis1	AAGGGAT	207	402.5	[100;800]	1.31	GIS1	-	1(1/0)	-
Hap4	CCAATCA	114	540	[100:700]	0.83	HAP4	HAP4	3(2/1)	Respiration (*p *< 10^-15^)
Ino4	CATGTGA	177	454	[100:1000]	1.24	INO4	INO4	1(0/1)	Lipid, fatty-acid and isoprenoid metabolism (*p *< 10^-5^)
Mbp1	ACGCGTC	23	225	[0;600]	3.25	MBP1	MBP1	29(18/11)	DNA synthesis and replication (*p *< 10^-11^)
Met31	TGTGGCG	302	424	[100;1000]	1.35	MET31	MET31	4(4/0)	-
Met4	CTGTGGC	362	500	[100;800]	1.08	MET4	MET4	1(1/0)	Amino acid metabolism (*p *< 10^-6^)
Msn2/4	AAAGGGG	49	332	[0;500]	1.92	MSN2/4	-	105(93/12)	-
Gln3	GATAAGA	143	434	[0;900]	1.23	-	-	7(7/0)	Nitrogen and sulfur metabolism (*p *< 10^-6^)
PAC	GCGATGAG	4	164.5	[0;400]	6.77	PAC	-	141(28/113)	rRNA transcription (*p *< 10^-10^)
Pdr3	CCGCGGA	357	378	[0;500]	2.34	PDR3	-	18(15/3)	-
Rap1	TGGGTGT	110	498.5	[100;900]	1.19	RAP1	-	13(1/12)	-
Reb1	CGGGTAA	1	213	[0;1000]	6.48	REB1	REB1	-	-
Rox1	AACAATAG	77	288.5	[0;500]	2.05	-	-	1 (0/1)*	-
Rpn4	TTTGCCACC	20	175.5	[0;800]	2.01	RPN4	-	10(10/0)	Cytoplasmic and nuclear degradation (*p *< 10^-31^)
RRPE	AAAAATTTT	2	188	[0;600]	3.04	RRPE	-	167(31/136)	rRNA transcription (*p *< 10^-16^)
Ste12	TGAAACA	282	477	100;1000]	1.15	STE12	STE12	5(3/2)	fungal cell differentiation (*p *< 10^-5^)
Sum1/Ndt80	TGACACA	51	385	[0;600]	1.32	SUM1	SUM1	1(1/0)	-
Swi4	CGCGAAA	19	261	[0;600]	3.25	SWI4	SWI4	39(22/17)	-
TATA	TATATAA	18	291	[100;700]	4.70	-	-	49(40/9)	-
Ume6	TAGCCGCC	6	457.5	-	3.92	UME6	-	-	Meiosis (*p *< 10^-7^)
Xbp1	CCTCGAG	219	348	[0;700]	2.41	XBP1	-	40(34/6)	-

For each known regulatory element, we show the best *k*-mer, its rank within the set of 398 highest-scoring *k*-mers, the median distance to ATG (for occurrences upstream of genes within the conserved set), the optimal window, the corrected ratio of upstream/coding bias, the best known motif (see Materials and methods), the best chromatin IP (ChIP) enrichment (see Materials and methods), the total (upregulated/downregulated) number of microarray conditions in which the *k*-mer was found (see Materials and methods), and the best MIPS enrichment. *This sequence was the most significantly over-represented 8-mer in the upstream regions of genes that were downregulated upon overexpression of the *Rox1 *gene (a known repressor of hypoxia-induced genes under aerobic conditions [95]), as part of a series of microarray experiments measuring *S. cerevisiae *transcriptional response to various stresses [96].

**Table 2:** Known regulatory elements obtained when applying FastCompare to *C. elegans *and *C. briggsae*

Sequence	Rank	D_ATG_	W_ATG_	Orientation	U/C	Experiment	TRANSFAC	Comments
TGATAAG	5	746	[0;600]	← (*p *< 10^-6^)	1.67	103(56/47)	GATA-1, GATA-2	Known GATA factor
AATCGAT	6	865.5	[0;1900]	-	1.00	14(2/12)	CDP, Clox	Similar to DRE, embryonic development (*p *< 10^-8^)
TGACTCAT	8	708	-	→ (*p *< 10^-4^)	1.40	-	AP-1, GCN4, NF-E2	Known AP-1 site
GTGTTTGC	9	383.5	[0;800]	-	2.44	-	-	Known forkhead-related activator 4
CACGTGG	16	935	-	-	0.73	12(9/3)	Myc/Max, PHO4, USF	Known Myc-Max site in *Drosophila*
AAGGTCA	22	882	[0;1400]	-	1.52	35(16/19)	ER, HNF-4	Known HRE
TGACGTC	32	858	[0;1700]	-	0.94	1(1/0)	CREB, ATF	Known CREB site
TGTCATCA	42	879	-	-	0.80	-	Skn-1	Known SKN-1 site
CAGCTGG	56	1093	[100;2000]	-	0.67	5(2/3)	AP-4, HEN-1	Known AP-4 and MyoD/CeMyoD site
AGAGAGA	57	893	-	→ (*p *< 10^-90^)	1.43	4(2/2)	-	Known GAGA-factor site
GTAAACA	79	818	[0;400]	-	2.69	28(28/0)	Freac, SRY	Known DAF-16 site
CCCGCCC	88	535	[0;1400]	-	2.48	1(0/1)	Sp1, GC box	Known Sp1 site
ATCAATCA	100	911	-	-	0.93	1(1/0)	Pbx-1	Known Pbx-1 site
CAGGTGA	111	845	[0;200]	-	2.25	-	Lmo2, RAV1	Known Snail site in *Drosophila*
TTCGCGC	148	651.5	[0;1200]	-	1.7	16(7/9)	E2F	Known E2F site, embryonic development (*p *< 10^-6^)

For each known regulatory element, we show the best *k*-mer, its rank within the set of 437 highest scoring *k*-mers, the median distance to ATG (for occurrences upstream of genes within the conserved set), the optimal window, the orientation bias, the corrected ratio of upstream/coding bias, the total (up-regulated/down-regulated) number of microarray conditions in which the *k*-mer was found (see Materials and methods), TRANSFAC matches, and the best GO enrichment.

**Table 3:** Novel predicted regulatory elements obtained when applying FastCompare to *C. elegans *and *C. briggsae*

Sequence	Rank	D_ATG_	W_ATG_	Orientation	U/C	Experiment	Comments
CTGCGTCT	1	635.5	-	→ (*p *< 10^-10^)	2.70	-	Unknown site
CGACACTCC	4	234	[0;1500]	-	2.49	-	Unknown site, positive regulation of growth (*p *< 10^-7^)
CTCCGCCC	14	440	[0;900]	-	3.51	2(2/0)	Unknown site, similar to Sp1
CGAGACC	20	738	[0;1900]	-	1.34	30(7/23)	Unknown site, embryonic development (*p *< 10^-7^)
CGCGACGC	23	457	[0;1900]	-	2.34	-	Unknown site
ATTTCGCAA	29	641	[0;1900]	-	2.50	1(0/1)	Unknown site
CGTAAATC	31	514	[0;600]	-	2.78	-	Unknown site
TTGCGGAC	39	253	[0;1700]	-	1.43	-	Unknown site
ATGATGCAA	44	600	[0;1600]	-	0.88	-	Unknown site
CGCGCTC	46	576	[0;900]	-	2.73	2(0/2)	Unknown site
TGGCGCC	49	770.5	[0;1800]	-	1.01	-	Unknown palindromic site
AACCGGTT	50	651	[0;1900]	-	1.41	-	Unknown palindromic site
TAAAGGCGC	61	524	[0;700]	-	8.67	27(12/15)	Unknown site
CGCGCGC	120	455	[0;600]	-	5.40	11(3/8)	Unknown site
CTAATCC	228	934	-	→ (*p *< 10^-7^)	1.20	-	Unknown homeodomain site, similar to Bicoid
TACCGTA	242	975	[0;500]	-	2.23	20(18/2)	Unknown site

*k*-mers shown here were selected from the list of 437 highest scoring *k*-mers based on their short median distance to ATG, short optimal window, significant orientation bias, strong over-representation ratio (U/C), presence in upstream regions of over/underexpressed genes in several microarray conditions, palindromicity or resemblance to known sites in other species.

**Table 4:** Known and novel predicted regulatory elements, obtained when applying FastCompare to *D. melanogaster *and *D. pseudoobscura*

Sequence	Rank	D_ATG_	W_ATG_	Orientation	U/C	TRANSFAC	Comments
**(a) **Known regulatory elements							
AACAGCTG	1	373	[0;1800]	-	1.64	-	Known AP-4/MyoD site
ATTTGCATA	3	882	[100;2000]	-	3.20	Oct-1	Known (mammalian) Oct-1 site
CACGTGC	5	825.5	-	-	1.02	Myc/Max, PHO4, USF	Known Myc/Max site
ATTTATGC	6	866	-	-	3.52	CdxA	Known CdxA site
TGACGTCA	9	825	-	-	2.36	CREB	Known CREB site
TGATAAG	11	760.5	[0;1100]	-	2.53	GATA	Known GATA site, carbohydrate metabolism (*p *< 10^-5^)
TATCGATA	12	168	[0;1900]	-	5.39	-	Known DRE site
TTTATGGC	14	978.5	-	-	2.82	Abd-B	Known Abd-B site
TAATTGA	24	907	[0;1900]	-	2.58	Ubx, Athb-1	Known Antp site
GAGAGAG	26	705.5	-	← (*p *< 10^-4^)	1.87	-	Known GAGA site, morphogenesis (*p *< 10^-23^)
CAGGTGC	33	1020.5	-	-	0.83	Sn	Known Snail site
TGACTCA	46	911	[100;2000]	-	1.89	AP-1, GCN4	Known AP-1 site
ATCAATCA	51	967	[0;1900]	-	1.72	Pbx-1	Known Pbx-1 site
AAGGTCA	93	1015.5	[400;1900]	-	1.16	HNF-4, ER	Known HRE
AACATGTG	105	994	[100;2000]	-	1.62	-	Known Twist site
GTAAACA	147	813	[0;1200]	-	2.54	Freac, SRY	Known DAF-16 site in *C. elegans*
							
**(b) **Novel predicted regulatory elements							
ACACACAC	2	922.5	-	→ (*p *< 10^-12^)	1.97	-	Unknown site, embryonic development (*p *< 10^-9^)
CAAGGAG	13	1091	[200;2000]	← (*p *< 10^-8^)	0.84	-	Unknown site
GCACACAC	29	886	-	-	1.80	-	Unknown site, histogenesis (*p *< 10^-5^)
CAAGTTCA	30	920	[0;1900]	-	1.23	-	Unknown site
TAATTAA	31	871	[500;2000]	-	3.07	Ftz	Unknown palindromic homeodomain-like site
CAACAACA	42	968.5	[200;2000]	-	1.22	-	Unknown site, regulation of transcription (*p *< 10^-5^)
TGGCGCC	48	951	-	-	0.84	-	Unknown palindromic site
CCTGTTGC	111	653	[0;1800]	-	0.90	-	Unknown site
GTGTGACC	112	296	[0;1900]	→ (*p *< 10^-5^)	2.22	-	Unknown site
CAGGTAG	143	924.5	[0;1700]	-	0.94	-	Unknown site, cell fate commitment (*p *< 10^-8^)
CACACGCA	145	968.5	-	-	1.49	-	Unknown site, cellular morphogenesis (*p *< 10^-5^)
GTCAACAA	169	904	-	-	1.48	-	Unknown site, similar to DAF-16
AAATGGCG	205	592	-	-	1.54	-	Unknown site
TTGACCCA	239	860	[0;1700]	-	1.60	-	Unknown site
TGACACAC	273	860	-	-	1.83	-	Unknown site
TGTCAAC	281	999	[100;1900]		1.55	-	Unknown site

**(a) **For each known regulatory element, we show the best *k*-mer, its rank within the set of 469 highest scoring *k*-mers, the median distance to ATG (for occurrences upstream of genes within the conserved set), the optimal window, the orientation bias, the corrected ratio of upstream/coding bias, the total (up-regulated/down-regulated) number of microarray conditions in which the *k*-mer was found (see Method), TRANSFAC matches, and the best GO enrichment. **(b) **Novel predicted regulatory elements. *k*-mers shown here were selected from the list of 469 highest scoring *k*-mers based on their short median distance to ATG, short optimal window, significant orientation bias, strong over-representation ratio (U/C), presence in upstream regions of over/underexpressed genes in several microarray conditions, palindromicity or ressemblance to known sites in other species.

**Table 5:** Known and novel predicted regulatory elements, obtained when applying FastCompare to *H. sapiens *and *M. musculus*

Sequence	Rank	D_ATG_	W_ATG_	Orientation	U/C	Experiment	TRANSFAC	Comments
**(a) **Known regulatory sequences								
CCCGCCC	1	256	-	-	2.26	8(7/1)	Sp1, GC box	Known Sp1 site, transcription from pol II promoter (*p *< 10^-5^)
GCCCCGCCC	2	165	-	-	4.64	9(9/0)	Sp1, GC box	Known Sp1 site, variant from above
CCGGAAG	4	160.5	[0;700]	-	2.37	-	Ets1, Elk1	Known Ets site, RNA metabolism (*p *< 10^-6^)
CACGTGAC	18	122.5	[0;600]	-	4.90	-	USF, GBP, SREBP-1	Known Myc/Max site
TGACGTCA	19	107	[0;1000]	-	4.24	-	CREB	Known CREB site
CGCATGCG	24	132	[0;1600]	-	4.26	-	-	Known palindromic octamer sequence (POS)
CCAATCAG	37	239	[0;700]	-	2.85	4(0/4)	NF-Y, CCAAT	Known CAAT box and CCAAT enhancer binding protein site
CGGAAGTGA	51	94	[0;1000]	-	3.96	-	STAT3	Known GA-binding protein (GAB) site
CCGCCTC	78	632	[0;500]	-	4.26	9(8/1)	-	Known insulin response element
CACGTGG	82	429.5	[0;300]	-	2.09	-	USF, Myc-Max	Known Myc/Max site, different from above
TAATCCCAG	119	1258	[100;2000]	← (*p *< 10^-14^)	7.06	3(1/2)	-	Similar to Bicoid (*Drosophila*), RNA processing (*p *< 10^-5^)
CACCTGC	227	925	[0;600]	-	1.64	1(1/0)	E47, Lmo2	Known ZEB site in vertebrates, Zfh-1 in *Drosophila*
ATTTGCAT	234	729	[0;300]	-	1.95	-	Oct-1	Known Oct-1 site, chromatin assembly/disassembly (*p *< 10^-8^)
CCAAGGTCA	242	801	[0;1800]	-	1.59	-	-	Known HRE site
GGAAGTCCC	253	124.5	[0;300]	-	2.60	-	NFκB	Known NFκB site
CAGCTGC	256	850	[0;1600]	-	1.03	-	AP-4, HEN1	Known AP-4, MyoD site
TTTCGCGC	275	245	-		2.42	-	E2F	Known E2F site
								
**(b) **Novel predicted regulatory sequences								
CGCAGGCGC	6	127	-	-	2.76	-	-	Unknown site
GCGCCGC	13	311	[0;1900]	← (*p *< 10^-5^)	1.41	-	-	Unknown site
TCTCGCGA	17	116	[0;1700]	-	4.45	-	StuAp	Unknown site, similar to E2F
TTAAAAA	52	1142	[100;2000]	-	2.19	21(0/21)	-	Unknown site
CTCCGCCC	60	242.5	[0;1300]	-	3.85	-	-	Unknown site, similar to Sp1
CCCCTCCC	67	563	[0;500]	→ (*p *< 10^-4^)	5.12	1(0/1)	-	Unknown site, regulation of transcription, DNA-dependent (*p *< 10^-5^)
AAGATGGCG	76	334	[0;1300]	-	1.14	-	-	Unknown site
CTGCGCA	89	199	[0;300]	-	3.63	-	-	Unknown site
CCAGCCTGG	123	1245	[200;2000]	-	4.42	-	-	Unknown site
CCTGCCC	162	788	[0;1800]	-	1.55	21(20/1)	E47/Sp1	Unknown site
CCCTTTAAG	166	230	[0;800]	→ (*p *< 10^-10^)	3.45	-	-	Unknown site
CCCCAGC	207	785	-	-	1.42	22(22/0)	-	Unknown site
TACAACTCC	225	154	[0;700]	-	2.51	-	-	Unknown site
GTGAGCCAC	248	1208	-	→ (*p *< 10^-6^)	6.28	-	-	Unknown site

**(a) **For each known regulatory element, we show the best *k*-mer, its rank within the set of 284 highest scoring *k*-mers, the median distance to ATG (for occurrences upstream of genes within the conserved set), the optimal window, the orientation bias, the corrected ratio of upstream/coding bias, the total (upregulated/downregulated) number of microarray conditions in which the *k*-mer was found (see Materials and methods), TRANSFAC matches, and the best GO enrichment. **(b) **Novel predicted regulatory elements. *k*-mers shown here were selected from the list of 284 highest-scoring *k*-mers based on their short median distance to ATG, short optimal window, significant orientation bias, strong over-representation ratio (U/C), presence in upstream regions of over/underexpressed genes in several microarray conditions, palindromicity or resemblance to known sites in other species.

**Table 6:** Numbers of 7-mers and 8-mers shared between the 400 highest scoring 7-mers and 8-mers, respectively, in each pair of phylogenetic groups considered

**(a) **7-mers				
	Y	W	F	M
Y	-	64(*p *< 10^-17^)	45(*p *< 10^-6^)	43(*p *< 10^-6^)
W		-	103(*p *< 10^-48^)	43(*p *< 10^-6^)
F			-	43(*p *< 10^-6^)
M				-
				
**(b) **8-mers				
	Y	W	F	M

Y	-	36(*p *< 10^-19^)	26(*p *< 10^-11^)	10(*p *= 0.025)
W		-	59(*p *< 10^-45^)	23(*p *< 10^-8^)
F			-	16(*p *< 10^-4^)
M				-

The number of **(a) **7-mers and **(b) **8-mers shared between the 400 highest scoring 7-mers and 8-mers, respectively, in each pair of phylogenetic groups considered. Y, yeasts; W, worms; F, flies; M, mammals. *p*-values were calculated using the hypergeometric distribution, as described in Materials and Methods.

**Table 7:** Comparison of conservation scores between highest scoring *k*-mers and position weight matrices (PWM) for 20 known regulatory elements in *S. cerevisiae*, obtained when comparing *S. cerevisiae *and *S. bayanus*

Name	Sequence	Score	PWM consensus	Score
Bas1	AAGAGTCA	93.8*	[AG][AG]NANGAGTCA	80.9
Cbf1	CACGTGA	421.3*	[AG][AG]TCACGTG	406.5
Fkh1/2	TAAACAA	110.3	GTAAACAA[AT]	114.1*
Gcn4	TGACTCA	93.4	[AG][AG]TGA[CG]TCA	135.4*
Gcr1	TGGAAGC	82.7*	[AG]GCTTCCT CG]T	42.7
Hap4	CCAATCA	104.2*	G[AG][AG]CCAATCA	96.6
Ino4	CATGTGA	91.2*	CAT[CG]TGAAAA	61.1
Mbp1	ACGCGTC	204.1	ACGCGTNA[AG]N	210.2*
Msn2/4	AAAGGGG	140.1	A[AG]GGGG	169.7*
PAC	GCGATGAG	404.6	GCGATGAGNT	520.3*
Pdr3	CCGCGGA	76.9	[CG]NNTCCG[CT]GGAA	102.5*
Rap1	TGGGTGT	103.8	[AG]TGTN[CT]GG[AG]TG	253.2*
Reb1	CGGGTAA	Inf	[CG]CGGGTAA[CT]	Inf
Rpn4	TTTGCCACC	218.6	GGTGGCAAAA	259.4*
RRPE	AAAAATTT	509.9*	TGAAAAATTT	388.80
Ste12	TGAAACA	81.4	ANNNTGAAACA	100.0*
Sum1/Ndt80	TGACACA	135.4*	[AG][CT]G[AT]CA[CG][AT]AA[AT]	100.0
Swi4	CGCGAAA	224.1*	NNNNC[AG]CGAAAA	116.6
Ume6	TAGCCGCC	377.2	TCGGCGGC[AT]A	410.0*
Xbp1	CCTCGAG	86.7	GCCTCGA[AG]G[AC]G[AG]	141.7*

*Indicates which regulatory element representation (*k*-mer or weight matrix) obtained the highest conservation score. Inf corresponds to very large conservation scores, obtained when taking the negative natural logarithm of near-zero hypergeometric p-values.
